# Transdiagnostic group cognitive behaviour therapy for anxiety in bipolar disorder—a pilot feasibility and acceptability study

**DOI:** 10.1186/s40814-020-00719-6

**Published:** 2020-11-07

**Authors:** Tania Perich, Philip B. Mitchell, Tanya Meade

**Affiliations:** 1 School of Psychology, Western Sydney University, Penrith, Australia; 2 School of Psychiatry, Western Sydney University, Sydney, Australia

**Keywords:** Bipolar disorder, Cognitive behaviour therapy, Group therapy, Anxiety

## Abstract

**Objective:**

Anxiety is prominent for many people living with bipolar disorder, yet the benefit of psychological interventions in treating this co-morbidity has been minimally explored and few studies have been conducted in a group format. This study aimed to assess the feasibility and acceptability of a transdiagnostic cognitive behaviour therapy group anxiety programme (CBTA-BD) for people living with bipolar disorder.

**Methods:**

Participants were recruited to take part in a 9-week group therapy programme designed to treat anxiety in bipolar disorder using cognitive behaviour therapy. They were assessed by structured interview (SCID-5 RV) to confirm the diagnosis of bipolar disorder and assessed for anxiety disorders. Self-report questionnaires—DASS (depression, anxiety, stress), ASRM (mania), STAI (state and trait anxiety) and Brief QOL.BD (quality of life) pre- and post-treatment were administered.

**Results:**

Fourteen participants enrolled in the programme, with 10 participants (5 male; 5 female) completing the follow-up assessments. Two groups (one during working hours, the other outside working hours) were conducted. The programme appeared acceptable and feasible with a mean of 6.9 (77%) sessions attended, though five (50%) participants completed less than 3 weeks homework.

**Conclusion:**

The transdiagnostic cognitive behaviour therapy group anxiety programme (CBTA-BD) proved feasible and acceptable for participants; however, homework compliance was poor. A larger randomised pilot study is needed to assess the benefits of the intervention on symptom measures and address homework adherence, possibly through providing support between sessions or tailoring it more specifically to participant needs.

## Introduction

Bipolar disorder affects approximately 1% of the population in Australia and around the world [1] and is defined by the presence of mania or hypomania symptoms along with periods of depression [2]. Depressive symptoms tend to dominate the course of the illness [3, 4], and people living with the condition may be symptomatic up to 56% of the time [5].

Anxiety is prominent for many people living with bipolar disorder and can occur as a manifestation of the disorder itself [2]. However, in addition, anxiety disorders are also highly comorbid with bipolar disorder with about 40% meeting the diagnostic criteria at any time [6], and about 35% of people with bipolar disorder also meet the criteria for a co-occurring anxiety disorder during euthymia [7]. The most common comorbid anxiety conditions are panic disorder, social anxiety and generalised anxiety disorders; however, multimorbidity is also common [6]. Co-morbid anxiety in bipolar disorder is associated with greater current symptom severity, increased impairment and increased risk of mood episodes [8] and lifetime suicide attempts [9]. Studies have also found that anxiety in bipolar disorder is also associated with lower quality of life, earlier relapse and diminished role function, with a greater impact also being found with the presence of multiple co-occurring anxiety disorders. Here, those with bipolar disorder and anxiety reported lower enjoyment and satisfaction in a range of daily task areas (quality of life) with lower scores reported for those who had more than one co-occurring anxiety disorder [10].

A systematic review of psychological intervention studies that had been conducted of global studies, mostly in high-income countries, identified 22 studies that measured anxiety in addition to hypo/mania and depression as part of the research outcomes [11]. They found preliminary evidence that anxiety scores significantly improved after cognitive behavioural therapy (CBT) for bipolar disorder [11]. However, despite measuring anxiety, no studies targeted the reduction of anxiety symptoms directly and only seven of those studies specifically targeted the treatment of an anxiety disorder, with PTSD being the most specifically targeted condition [11]. Furthermore, the most common co-morbid anxiety condition—social anxiety disorder—was not addressed in any study, while the second most common—generalized anxiety disorder—was addressed in only one study (that comprised four participants) [11].

A recent pilot study with 29 subjects has explored the use of a psychological intervention designed specifically for anxiety for people with bipolar disorder using a transdiagnostic framework, the Unified Protocol, which addresses features common to a range of emotional disorders [12]. Here, rather than a specific disorder being addressed, this protocol was developed for emotional disorders broadly, aiming to teach participants approaches to emotions that may be applied to a range of situations incorporating elements of mindfulness and cognitive behaviour therapy. The authors examined the impact of 18 one-to-one sessions of a transdiagnostic CBT compared to treatment as usual for people with bipolar disorder and a co-occurring anxiety disorder The transdiagnostic therapy targeted key features common to all anxiety disorders rather than identifying a specific disorder. The researchers found that anxiety symptom scores significantly decreased, along with depression, as a result of the intervention, showing promise for the role of transdiagnostic interventions in treating anxiety comorbid with bipolar disorder. Transdiagnostic approaches may be particularly relevant for treating anxiety in bipolar disorder, given the high levels of co-morbidity between anxiety disorders, or multimorbidity, noted in this population [7], and also given that anxiety may occur as part of the condition itself [2].

Although some previous group therapy programme studies have assessed anxiety outcomes in bipolar disorder [13], very little is known about the efficacy of psychological interventions in treating co-occurring anxiety specifically for people living with bipolar disorder, and few, if any, studies have been conducted in a group format. The study reported here aimed to pilot a transdiagnostic group cognitive behaviour therapy anxiety programme for people living with bipolar disorder, specifically aiming to assess the feasibility and acceptability of the programme and monitor drop-out rates, weekly attendance and homework compliance. It was hypothesised that the programme would be both feasible and acceptable for participants with bipolar disorder.

## Method

### Participants

Participants were recruited from the community via Facebook advertising broadly targeting people living in the area of Western Sydney, New South Wales, Australia. The inclusion criteria were a confirmed diagnosis of bipolar disorder, under the care of a GP or psychiatrist, not experiencing a current episode of either depression or hypo/mania and aged over 18 years.

Thirty-two participants expressed interest in taking part in the study through the completion of an online form. Of these, 23 (74%) expressed a preference for after-hours or weekend group sessions. Of those who expressed an interest in the study, eight did not reply to the initial follow-up email or phone call and one reported that they had been recently hospitalised. Seven reported that they were unable to attend due to work commitments, travel or other obligations.

The remaining 14 participants completed all baseline measures and were allocated to one of the two groups. One group contained six participants, who indicated that they were able to take part in sessions during business hours. The other group consisted of eight participants whose preference was for sessions held on weekends. The study was approved by the Western Sydney University Human Research Ethics Committee (H12690).

### Measures

#### Clinician-administered

Structured Clinical Interview for DSM-5–Research Version (SCID-5-RV) [14]. The SCID-5-RV is a semi-structured clinical interview schedule designed for research studies which assesses diagnostic criteria for major psychiatric disorders as defined in the DSM-5 and can be adapted for each research study. Modules relevant to bipolar disorder (mania, hypomania, depression) and modules relating to anxiety disorders were used. These included panic disorder, agoraphobia, simple phobia, social anxiety disorder and generalised anxiety disorder modules. The diagnostic reliability and validity of the instrument are not yet available for Australian samples; however, earlier research on earlier versions of the SCID-I has indicated bipolar disorder interrater reliabilities as a median kappa of 0.74, indicating a good level of agreement between raters [15].

### Self-report measures

#### Demographics

Demographic questions included age, marital status, gender, country of birth, education and employment. Questions regarding clinical course were also asked, including illness course, age of diagnosis, number of prior episodes and previous hospitalisations.

#### Depression, Anxiety and Stress Scales [16]

The Depression, Anxiety and Stress Scales (DASS-21) is a 21-item self-report scale that assesses symptoms of depression, anxiety and stress. It contains items such as ‘I was worried about situations in which I might panic and make a fool of myself’. Participants indicate responses from 0 ‘did not apply to me at all’ to 3 ‘applied to me very much, or most of the time’. The range on the subscales is from 0 to 42 with higher scores indicating higher levels of depression, anxiety and stress. The DASS-21 has been found to have good reliability for all three subscales (*α* = 0.91 ‘depression’; *α* = 0.81 for ‘anxiety’; *α* = 0.89 for ‘stress’) in Australian samples [17].

#### State/Trait Anxiety Inventory [18]

The State/Trait Anxiety Inventory (STAI) measures both state and trait anxiety using two 20-item subscales, one which asks participants how they feel ‘right now’ (state anxiety) and the other, how they feel ‘generally’ (trait anxiety). Items include ‘I feel at ease’ where participants indicate from 1 ‘not at all’ to 4 ‘very much so’. The range on the subscales is from 20 to 80 with higher scores indicating higher levels of anxiety. The STAI has good internal consistency (state anxiety *α* = .95; trait anxiety *α* = .96) in Australian samples [19].

#### Altman Self-Rating Mania Scale [20]

The Altman Self-Rating Mania Scale (ASRM) is a 5-item self-report scale that assesses manic symptoms during the past week. It contains items such as ‘I feel happier or more cheerful than usual all of the time’ and is scored from 0 (absent) to 4 (present to a severe degree). Scores range from 0 to 16 with higher scores indicating higher levels of hypo/mania symptoms. It has a good reliability with *α* = 0.79 in US samples [21].

#### Brief version of Quality of Life in Bipolar Disorder [21]

The Brief version of Quality of Life in Bipolar Disorder (Brief QOL.BD) is a 12-item measure derived from the larger 56-item measure that asks about participation in quality of life-related activities over the previous 7 days. Subscales include physical, sleep, mood, cognition, leisure, social, spirituality, finance, household, self-esteem, independence and identity. Participants rate items from ‘strongly disagree’ to ‘strongly agree’*.* It includes items such as ‘been interested in my social relationships’. The Cronbach alpha for this scale has been reported as ranging from *α* = 0.87 to 0.89, indicating good reliability in Canadian samples [21].

### Procedure

The study was advertised via Facebook, which provided a link to the study’s participant information sheet, online consent form and then a preliminary assessment of eligibility (under the care of a GP or psychiatrist, over 18 and previously diagnosed with bipolar disorder). Upon receipt of the online information and indicating that they consented to take part in the study, potential participants provided contact details and were contacted via phone to ascertain their clinical status. All participants provided written consent to participate by ticking the consent box online; however, those participants who did not respond to contact requests after providing consent were considered as no longer wishing to participate in the study. Participants took part in a telephone interview confirming their diagnosis of bipolar disorder and whether they currently meet the criteria for a mood episode using the SCID 5 (research version) [14]. Any current or lifetime anxiety disorder diagnoses were also assessed (panic disorder, agoraphobia, simple phobia, social anxiety disorder and generalised anxiety disorder) [14].

After completion of the interview and confirmation of the diagnosis and current mood status, participants were asked to complete online questionnaires that asked about demographics features of their illness, followed by self-report questionnaires on anxiety and mood symptoms (DASS, STAI, ASRM) and quality of life (Brief QoL.BD).

Participants then attended a 9-week group therapy programme designed to treat anxiety using CBT. After completion of the 9-week programme, participants completed the same pre-programme battery of self-report questionnaires. They were also invited to participate in a qualitative interview conducted by a research assistant regarding their experiences of the programme and content. This included questions such as ‘In thinking about your experience of the group, what about the programme had the most impact on your anxiety?’, ‘Which aspects of the programme did you find helpful?’, ‘Which did you find least helpful?’ and ‘Tell me more about what happened, some examples, or what was done in the programme that helped?’. A thematic analysis of the qualitative data regarding the group process and structure is presented elsewhere in more detail.

### Intervention

The programme was developed using standard CBT elements commonly used in a wide range of individual-based CBT anxiety treatment programmes with elements specifically enhanced for people living with bipolar disorder, such as highlighting the role that mood shifts may play in anxiety symptom severity. The group programme was conducted at Western Sydney University, Penrith Campus, with each session lasting for between 1.5 and 2 h. The 9-session programme involved psychoeducation, de-arousal strategies and other techniques, self-management planning and targeting of cognitive beliefs about anxiety. The techniques were taught in a manner where they were encouraged to apply these in a range of settings and situations (e.g. social, agoraphobia situations), and participants were encouraged to generate their own examples of when and how they would apply the techniques. The impact of depression and hypo/mania on anxiety was discussed, and participants were encouraged to discuss bipolar-specific experiences. A session-by-session outline of the programme is listed in Table 1.

**Table 1:** Description of the content of the CBT programme

Session	Content description
Session 1: Introduction to the programme	Introduce the CBT model, flight or fight response. Discussion of these components in relation to BD, education about different types of anxiety in BD and how it may vary depending on the mood state
Session 2: Relaxation training	Practice and describe the different types of relaxation training, progressive muscle relaxation activity, controlled breathing practice and other forms of relaxation for BD
Session 3: Anxiety and the importance of thoughts	Adaptive basis of the components of anxiety, explanation of automatic thoughts in anxiety, different types of classic thinking errors that can occur, discuss in a group how these may be different in mania and depression with BD
Session 4: Challenging anxious thoughts	Define worry and its role in maintaining anxiety, introduce the idea of challenging automatic thoughts, asking and answering disputing questions and constructing rational responses
Session 5: Behavioural experiments	Negative predictions and how they can impact on anxiety; constructing behavioural experiments, what behavioural experiments are and how they are important in treating anxiety; and discuss timing and mood states in BD
Session 6: Graded exposure	Avoidance and safety behaviours how it maintains anxiety in BD, describe what graded exposure is and its role in treating anxiety and describe exposure stepladders and how they are important in countering avoidance and safety behaviours
Session 7: Problem-solving	Problem-solving and the relationship between this and worry solvable worries and unsolvable worries and different steps in problem-solving: identify and define the problem, generate possible solutions/options list and evaluate alternatives and implement plan
Session 8: Assertiveness	Assertiveness and how this can be related to anxiety; talk about the differences in non-assertive, assertive and aggressive responses and protective skills when using healthy assertion
Session 9: Conclusion	Revision, plan for continuing to manage anxiety and BD and discuss potential stressors that may arise when implementing the plan and how they should be managed

Participants were allocated weekly homework for eight of the nine sessions which related to the content of the group session that week. Homework included elements from the sessions, such as progressive muscle relaxation practice, identifying cognitive distortions and cognitive restructuring. Sessions were conducted by a registered psychologist and attended by a research assistant who collected attendance and homework compliance data and recorded the content and group discussions for each session.

### Data analyses

Data was analysed using SPSS V 25. Descriptive mean score calculations were conducted for continuous self-reported measures used in the study (DASS, STAI, ASRM) and quality of life (Brief QOL.BD). Statistical analyses to determine significant differences were not conducted due to the small sample size and the pilot nature of the project.

## Results

Fourteen participants were assigned to the groups, with 10 participants (5 male; 5 female) completing all aspects of the baseline assessment, group therapy programme and post-treatment assessments. The mean age of this sample was 44.9 years (range 23–73 years; SD 1.1) with seven (70%) meeting the diagnostic criteria for one current DSM-5 anxiety disorder and three (30%) criteria for more than one anxiety disorder. Further demographic and clinical characteristics of the sample are presented in Table 2.

**Table 2:** Demographic and clinical characteristics of the completer sample

	Number	Per cent
Bipolar type		
Bipolar I	6	60
Bipolar II	4	40
Anxiety disorders co-morbidity		
Agoraphobia	2	20
Panic	1	10
Generalised anxiety disorder	3	30
Social anxiety disorder	4	40
Marital status		
Married/de facto	3	30
Divorced/separated	2	20
Widowed	1	10
Never married	4	40
Country of birth		
Australia	7	70
Bangladesh	1	10
England	1	10
Ireland	1	10
Medication use		
Olanzapine	3	30
Lithium	2	20
Epilim	2	20
Lamotrigine	2	20
Others	2	20
None	1	10

Drop-out rates were similar between the groups, with two participants dropping out of the first group (33%) (with one dropping out prior to commencement; 1 male and 1 female) and two (25%) for the second group (2 males). The attrition rate overall was four of 14 participants (29%). Excluding the participant who did not attend any sessions, the retention rate was 79%. Self-reported reasons for dropping out included transport problems (*n* = 1) and illness and increased depression symptomatology (*n* = 1), with two participants not reporting a specific reason for drop out (both did not reply to follow-up emails for post-group assessment).

Four participants completed the first group and six completed the second. The mean number of sessions attended was 6.9 (SD 1.1; range 6–9 sessions). Attendance rates were similar between the groups with group 1 participants attending a mean of 6.5 sessions (SD 1) and group 2 participants a mean of 7.17 sessions (SD 1.17). Five participants completed 6 sessions, and 5 completed 7 or more.

Of the homework assessed, 32.5% of the homework that was allocated was completed in the overall sample. Weekly homework was reported as being undertaken for up to six sessions of a possible eight (range 0–6) by participants. The mean weekly rate of homework compliance was 2.6 (SD 1.67) weeks. Five participants (50%) completed homework for two or less weeks, with five completing three or more weeks homework. Reasons recorded for not completing homework included being too symptomatic, not having attended the previous session or having trouble with applying the instructions to the situations as required. Two participants reported that they completed goals set from their homework after the completion of the 9-week programme, when they were better able to find more opportunities to apply the skills.

No serious adverse events were reported during the programme in response to the intervention; however, one participant became increasingly symptomatic after commencing the group programme. This was reported to the psychologist who facilitated the sessions as related to external life stressors, which escalated throughout the duration of the programme.

### Symptom measures and quality of life

Participants’ mean self-reported symptom scores at baseline and post-treatment for the DASS (depression, anxiety, stress), ASRM (mania) and STAI (state and trait anxiety) are reported in Table 3 for descriptive purposes. Significance tests were not conducted due to the small sample size. Descriptive pre- and post-scores on the quality of life subscales of the Brief QOL.BD physical, sleep, mood, cognition, leisure, social, spirituality, finance, household, self-esteem, independence and identity are also reported in Table 3.

**Table 3:** Symptom and quality of life measures

Measure	Pre-treatment		Post-treatment	
	Mean	SD	Mean	SD
DASS				
Depression	33.4	9.3	28.8	11.75
Anxiety	28.6	10.67	29	9.49
Stress	37	10.93	32.4	12.07
AMRS				
Hypo/mania	5.6	3.84	5.9	4.07
STAI				
Trait anxiety	56	15.83	48.9	13.7
State anxiety	43.4	18.66	42.4	15.56
BD.QOL				
Physical	3	0.94	2.8	1.23
Sleep	2.6	1.17	2.7	1.42
Mood	2.9	1.45	3.4	0.97
Cognition	2.4	1.08	2.5	1.4
Leisure	3	1.25	3.3	1.42
Social	2.6	1.35	2.8	1.23
Spirituality	2.6	0.97	2.9	1.2
Finance	2.9	1.37	3.8	1.03
Household	1.9	1.29	2.3	1.25
Self-esteem	2.7	1.57	3.2	1.23
Independence	4.1	1.29	4	1.16
Identity	2.7	1.64	3	1.63

Descriptive qualitative data of content aspects of the programme that were discussed by participants are presented in Table 4. Participants mentioned the relaxation techniques most often (*n* = 10), with all participants talking about this aspect of the programme and most finding this helpful overall. Thinking styles/cognitive distortions were also mentioned by most participants (*n* = 7), with the majority finding this helpful also. Aspects that were reported as unhelpful by some participants included relaxation and problem-solving (see Table 4).

**Table 4:** Content descriptions of the programme by participants

	Number^a^	Helpful/useful	Unhelpful/difficult	Quote
Relaxation techniques	10	8	2	“....it was really good just to feel physically relaxed and then I think the feeling of being – the feeling of my body being physically relaxed transferred a little bit to my mental state, being relaxed as well, like it’s sort of a connection between the – well, not sort of – I know that there’s a connection between our physical state or physical symptoms and our mental states as well.”
Cognitive restructuring	7	7	0	“And I had all of them and I thought, “Oh, I didn’t realise that I had these thoughts on a regular basis all the time.” They played out your thoughts that you had during that week and I was like, “Oh, is that nearly all of them?” and I didn’t realise they were unhelpful thoughts.”
Homework	7	3	4	“I was pretty slack with my homework tasks, to be honest. And I was feeling really guilty and anxious about that …So I did a little bit of it. I didn’t really do any of the relaxation or anything like that. I just found that difficult to find the time, really.”
Assertiveness	4	4	0	“And so, that’s helped me because I always used to say yes to everything even though I don’t want to. So, it’s helped me to be more mindful of what I actually really want rather than just try to please everyone.”
Problem-solving	3	2	1	“...it’s hard to solve a problem sometimes, you know what I mean? It’s all right to be given a set of rules, but it doesn’t work….no, I didn’t find that real helpful”
Behavioural experiments	3	2	1	“….you work your way up to things that would’ve ‘caused me more anxiety in the past and it’s like you get gradually used to it. I thought that was a really good – I found that idea and the stuff around that really, really useful”
Graded exposure	3	2	1	“…but still useful was the related exposure things, although with my particular issue, I find them a little bit hard to sort of work out how it’ll fit in, but I wanna work with it.”

^a^Number of participants who mentioned this content in their qualitative interview

## Discussion

This study describes a transdiagnostic group therapy programme using cognitive behaviour therapy specifically targeting anxiety for people living with bipolar disorder. The programme appeared acceptable and feasible for participants overall with high attendance rates noted throughout, with a minimum of six sessions being attended by participants who completed the post-treatment assessments. Participants described much of the content as being helpful; however, participants reported difficulty with homework completion, suggesting that this aspect of the programme was not acceptable. Results are consistent with other published studies of group therapy programmes, which have found these types of interventions are acceptable for participants and families [22, 23].

Attendance rates were comparable between the groups, with the group that was conducted during business hours and the one conducted during the weekend being attended at similar rates. However, greater initial interest in participation was expressed in the after-hours groups, with a larger group being recruited for the Saturday session than for the day session during the working week. This may suggest that feasibility was greater for the group conducted outside of business hours and that this may be a more viable option for participants to attend, especially for those who work full-time. Future programmes should consider how to support people living with bipolar disorder who work full-time and how programmes may be offered to accommodate such participants in health care settings.

The retention rate in this programme was found to be higher than other bipolar disorder anxiety-specific therapy programmes reported in previous studies. For example, Ellard et al. [12] found for their individual session programme for anxiety in bipolar disorder that the total attrition rate was 38%, compared to 29% in our study. However, the retention rate findings in the current study may be lower than other group studies. In one of the few previous studies that have conducted a group therapy programme for anxiety which included participants with bipolar disorder (treating PTSD specifically), retention rates were reported as 86% [24]. The reason for the wide range of retention rates are not explored here; however, it may reflect the population mix with Rosenberg et al.’s study containing only five participants who were diagnosed with bipolar disorder (with the majority having unipolar depression). More research is needed to explore if specific illness features of bipolar disorder may contribute to retention rates and how programmes may better accommodate these illness features in their design and delivery if this is the case.

Although homework is a central part of CBT, poor compliance was noted in the sample with less than half of participants reporting any form of homework completion. Some participants did not complete any of the homework assigned, and there were no participants who completed all the homework requirements every week. Future studies should consider either minimising homework or addressing some issues that may be associated with poor homework compliance, such as providing support between sessions.

Given that previous research has found that the quality of the homework may be as important as the quantity [25], it may be that less homework may produce better outcomes and that tailoring homework to participants based on their individual needs may also lead to greater compliance and better outcomes [25, 26]. Given that two participants completed the homework at a later stage, appropriate timing of the homework should also be considered when designing future interventions. A needs assessment of homework prior group may be required to enhance outcomes and compliance [27].

Limitations—in addition to the small sample size—included the SCID-5 RV not being administered post-treatment to assess whether the participants still met the criteria for specific anxiety disorders, even though bipolar disorder diagnosis and anxiety disorders were assessed at baseline. Although the study was designed to reduce dimensional anxiety across anxiety disorders, it would be beneficial to assess if categorical criteria would still be met following the intervention. A reasonably large percentage of the sample met the diagnostic criteria for more than one anxiety disorder prior to study entry. Further research could explore if the transdiagnostic approach is particularly useful for this group of participants and to address gaps that may not have been addressed by the intervention.

In conclusion, the pilot group therapy programme appeared feasible and acceptable for participants with bipolar disorder. Greater feasibility was observed for the group conducted after hours, with a higher number of participants expressing interest and enrolling in this group; however, retention rates were similar overall. Issues surrounding homework compliance should also be addressed, given the low compliance rates and also given that this is a central feature of successful CBT interventions. A larger-scale randomised-controlled pilot study should be conducted to assess the benefits of the intervention using a larger sample size that may provide more information about whether this may be effective in reducing anxiety in this population, given that feasibility and acceptability have been determined.

## Data Availability

The datasets generated and/or analysed during the current study are not publicly available as consent was not obtained for this purpose but are available from the corresponding author on reasonable request.
