# Correction to: Transdiagnostic group cognitive behaviour therapy for anxiety in bipolar disorder—a pilot feasibility and acceptability study

**DOI:** 10.1186/s40814-020-00730-x

**Published:** 2020-11-24

**Authors:** Tania Perich, Philip B. Mitchell, Tanya Meade

**Affiliations:** 1 School of Psychology, Western Sydney University, Sydney, Australia; 2 School of Psychiatry, University of New South Wales, Sydney, Australia

**Correction to: Pilot Feasibility Stud 6, 170 (2020)**

**https://doi.org/10.1186/s40814-020-00719-6**

Following publication of the original article [1], the authors reported an error in the affiliations. The correct affiliations are presented below.

1. School of Psychology, Western Sydney University, Sydney, Australia

2. School of Psychiatry, University of New South Wales, Sydney, Australia

The original article has been corrected.
